# A Novel Alveolar Distractor Incorporating Nickel–Titanium Alloy Springs: A Preliminary In Vitro Study

**DOI:** 10.3390/ma15155151

**Published:** 2022-07-25

**Authors:** Sarun Chancharoen, Peerapong Santiwong, Dutmanee Seriwatanachai, Anak Khantachawana, Rochaya Chintavalakorn

**Affiliations:** 1 Department of Orthodontics, Faculty of Dentistry, Mahidol University, Bangkok 10400, Thailand; pump_jeen@hotmail.com (S.C.); peerapong.san@mahidol.ac.th (P.S.); 2 Department of Oral Biology, Faculty of Dentistry, Mahidol University, Bangkok 10400, Thailand; dutmanee.ser@mahidol.ac.th; 3 Department of Mechanical Engineering, Faculty of Engineering, King Mongkut’s University of Technology, Bangkok 10140, Thailand; anak.kha@kmutt.ac.th

**Keywords:** alveolar distraction osteogenesis, nickel–titanium, open-coil springs

## Abstract

A new design of an alveolar distractor using nickel–titanium (NiTi) open-coil springs was developed and investigated to produce distraction forces against the tensile forces of porcine attached gingiva to simulate human gingiva. We subjected 15 mm long NiTi open-coil springs (Highland and ORMCO) with three levels of forces (light, medium and heavy) to mechanical testing in a 37 ± 1 °C water bath. Ten strips of porcine mandibular attached gingiva were subjected to tensile tests to determine the resistance force. The forces from the springs were compared with the tensile forces from the porcine attached gingiva. Data between groups were analyzed with independent-samples *T*-tests (*p*-value < 0.05). The tensile strength and the Young modulus were greater in buccal compared to lingual porcine attached gingiva. Compared to other spring dimensions and companies, forces generated from 0.014 × 0.036″ ORMCO springs were the highest and could overcome the tensile resistance from porcine attached gingiva over the longest distraction range of 1.6 mm. This preliminary in vitro study introduced a new design of an alveolar distractor incorporated with NiTi open-coil springs that could generate light and continuous forces to overcome the resistance from porcine attached gingiva.

## 1. Introduction

Distraction osteogenesis (DO) is a tissue engineering technique for the progressive lengthening of bone. Intentional osteotomy is performed to split the bony segments before a tractional force is gradually applied across the osteotomized gap to promote new bone formation and soft tissue adaptation [1,2].

DO was first applied in the field of orthopedics for the lengthening of bones in the extremities before being adapted to various craniofacial regions to correct craniofacial anomalies [3]. Alveolar bone augmentation by vertical osteogenic distraction to increase the height of alveolar bone was first reported by Chin and Toth in 1996 [4].

As the forces of alveolar bone distraction are subject to opposing resistance from the surrounding soft tissues including the periosteum, gingiva, and oral mucosa, adequate levels of force created by the distractor are needed to overcome this to produce and maintain bone separation [5]. To determine the threshold of force required for DO, an understanding of the biomechanical behavior of human oral soft tissues is necessary. To achieve this, animal studies using porcine oral soft tissues have been used as a representative model system due to the biological and morphological similarities [6].

A conventional distractor consists of a threaded rod or screw which can be activated by the patients themselves by rotating the screw once or twice daily to produce a rate of bone displacement of 1 to 2 mm per day [7]. This, however, commonly leads to patient-related errors in DO such as activating the device in the wrong direction or inadequate activation due to patient discomfort [8,9]. Major complications related to technical failure such as patient compliance and device failure are generally more severe as compared to those related to abnormal physiological bone response such as premature consolidation and fibrous non-union [10]. Therefore, patient compliance in the distraction process is crucial towards a successful treatment outcome [11].

If the dependence on patient compliance is to be removed to improve the outcomes of DO, one of the strategies would be to update the design of the device using advancements in biomaterial technology. The concept of our study is to convert the distractor system from relying on manual activation to become an automatically activated system by utilizing the unique behavior of nickel–titanium (NiTi) alloys. Their distinct properties include superelasticity and shape memory, which are desirable in producing a constant and continuous distraction force over a long range of activation [12,13].

Superelastic NiTi open-coil springs have been commonly used in fixed appliance orthodontic treatment to create space between teeth. Their performance has been attributed to the austenite to martensite phase transformation, which occurs when tensile stress is applied [14]. When the energy stored from the stress-induced martensite phase is released during deactivation, a relatively constant force is produced during the martensite to austenite phase transformation. This behavior is exhibited by a plateau in the load–deflection curve of NiTi alloys. Moreover, the NiTi springs exhibit shape memory, which involves a return to their original shape after mechanical deformation due to a change from martensite to austenite induced by an increase in temperature [15,16].

Hence, it was in our interest to develop a novel design of the alveolar distractor using commercially available superelastic NiTi open-coil springs. This design would be able to automatically generate a continuous distraction force from springs that could eliminate the reliance on patient compliance for device activation, as the threated rod that requires patient cooperation for activation would be removed and hence potentially increase the treatment success rate. Our study aimed to investigate the mechanical properties of our novel distraction device by conducting tensile tests of two different manufacturers of open-coil springs within the distractor and their correlation with the resistance to the traction force of a porcine oral mucosa to simulate the distraction forces in human oral soft tissues.

## 2. Materials and Methods

### 2.1. Design of a Novel Alveolar Distractor

A novel distractor device was invented to be an automatic continuous distractor using superelastic NiTi open-coil springs exerting a continuous force. A material of choice for biomedical applications is titanium grade IV as it exhibits excellent biocompatibility, good formability and weldability, high strength, and low electrical conductivity from the formation of a thin passive oxide layer that gives a high resistance to corrosion [17].

**Figure 1:**
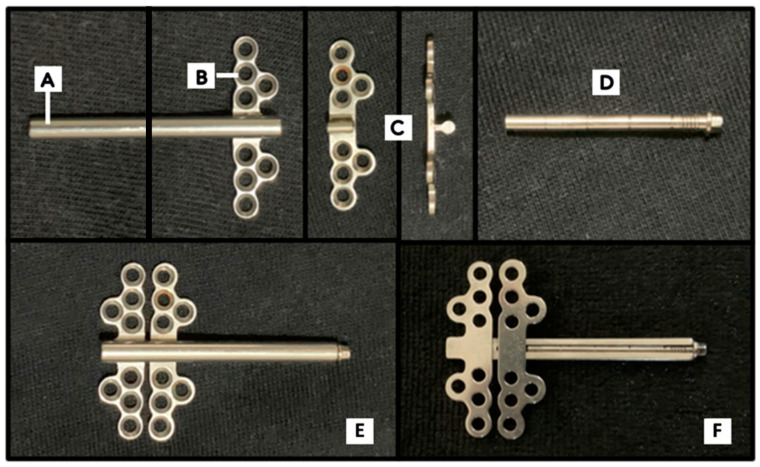
Components of the novel alveolar distractor: (**A**) distraction tube; (**B**) fixed base plate; (**C**) moveable base plate; (**D**) holding rod. The fully assembled distractor: (**E**) front view; (**F**) back view.

The components of our novel alveolar distractor were divided into four major parts, which were the distraction tube, the fixed base plate, the movable base plate, and the holding rod, as shown in Figure 1.

**Figure 2:**
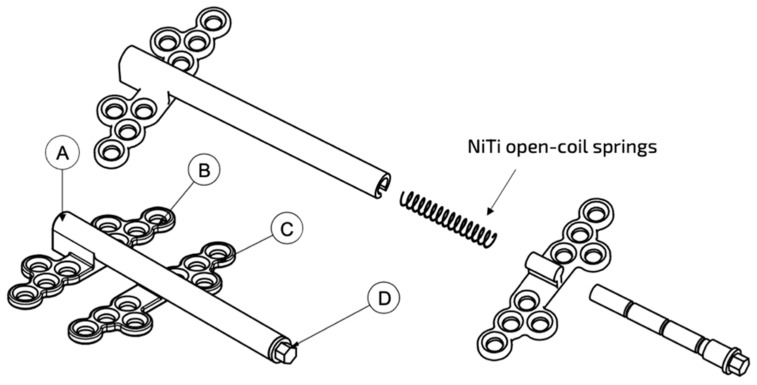
A drawing design showed the function of a new prototype of an alveolar distractor: (A) distraction tube; (B) fixed base plate; (C) moveable base plate; (D) holding rod.

After the NiTi spring is inserted into the distraction tube, the holding rod compresses or activates the spring against the moveable base plate (Figure 2). The thread head of rod is turned to lock with the tip of the distraction tube. The device is placed in this configuration during the surgical osteotomy and left as such throughout the latency phase of DO. To start the activation phase, the holding rod will be unlocked and rotated outwards to be cut at a length equal to the desired distraction distance. The shortened rod creates a space within the distraction tube, allowing the NiTi spring to generate an unloading force against the moveable plate. This causes the moveable plate to gradually slide along the distraction tube away from the fixed plate up to the desired distraction distance without the need for patient compliance. In the actual clinical environment, the unloading force of the spring will face resistance from the surrounding tissues.

### 2.2. Uniaxial Tensile Testing of Porcine Attached Gingiva

**Figure 3:**
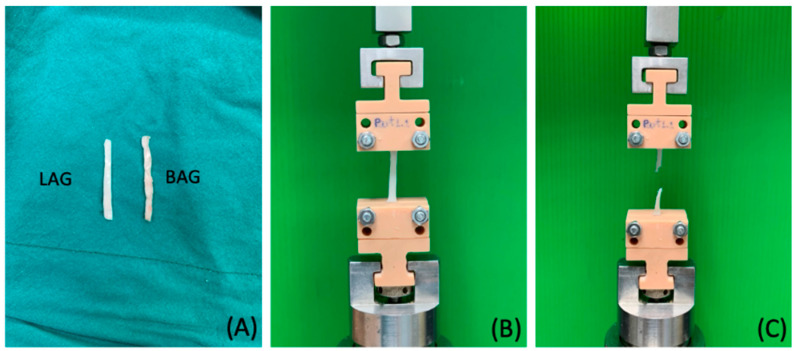
(**A**) Dissected buccal and lingual porcine attached gingiva; (**B**) experimental setup of the specimen and grips at the start of the tensile test; (**C**) failure of the specimen after tensile testing.

To simulate the gingival response during DO, specimens of buccal attached gingiva (BAG) and lingual attached gingiva (LAG) were collected from five porcine jaws within 24 h of animal sacrifice. Specimen templates of 3 mm × 30 mm were drawn on the buccal and lingual surfaces of the attached gingiva in the anteroposterior direction of the mandibular body and excised by using a No.5 surgical blade. A periosteal elevator was used to detach the attached gingiva to include all layers from the epithelium to periosteum (Figure 3A).

A total of five specimens from each buccal and lingual side were collected, immediately stored in normal saline (0.9% sodium chloride) and prepared for tensile testing within the same day of harvesting. The thickness of the specimens was measured at five areas each with an electronic digital caliper (Mitutoyo, Japan) to calculate the average thickness before testing [6].

The extension of the soft tissue during distraction was simulated by uniaxial tensile testing in a universal testing machine (Instron 5566, DT-40-073, Instron Ltd., Buckinghamshire, UK) equipped with a static load cell of 100 N at room temperature (23–25 °C). Custom-made grips were fabricated to hold the specimens vertically, with a fixed lower grip and a movable upper grip. The extension rate was set at 5 mm/min with a 0.003 N preloaded baseline until the specimens were torn apart (Figure 3B,C). The failure load was recorded from the load–deflection curve. The tensile strength and the Young modulus were calculated from the stress–strain curve using the formula: stress (σ) = force (F)/area, where A_o_ is the cross-sectional area perpendicular to the direction of the tensile force and strain (ε) = change in length (L_1_ − L_0_)/initial length (L_0_).

### 2.3. Mechanical Testing of the Novel Alveolar Distractor

A total of sixty commercial NiTi open-coil springs from two different manufacturers (Highland and ORMCO) in three force levels each were tested (Table 1).

**Figure 4:**
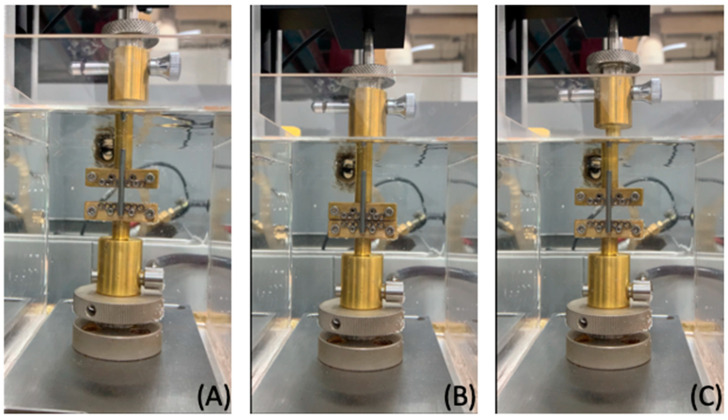
Experimental setup of the novel alveolar distractor for the compression–extension test. (**A**) Device configuration at the beginning of the experiment; (**B**) the upper grip compressing the springs downwards over 9.1 mm until the fixed and movable base plates were in contact; (**C**) the upper grip released upwards to the start position.

All springs, which were initially made of austenite, were cut to a length of 15 mm. A universal testing machine (Mecmesin’s MultiTest 2.5-I, PPT Group UK Ltd, England and Wale, UK) with a static load cell of 25 N was used for compression–extension tests for all specimens in an acrylic water bath fitted with a circulation pump and heating thermostat (Alpha, LAUDA DR. R. WOBSER GMBH & CO. KG, Lauda–Königshofen, Germany) to maintain a temperature of 37 ± 1 °C as measured by glass thermometer (SK Sato, Tokyo, Japan) (Figure 4A).

Five springs each from different manufacturers and force levels were selected and threaded onto a cylindrical rod (0.9 mm in diameter and 70 mm in length) to test the force generated when compressed to 55% of coil compression (8 mm) at a crosshead speed of 10 mm/minute and then gradually released to return to their original length at a rate of 2 mm/minute.

The remaining specimens were inserted into the distraction tube of the novel alveolar distractor, which was held vertically in place by custom-made grips, with the bottom grip holding the fixed base plate and the top grip holding the moveable base plate.

The springs were compressed from their initial length (15 mm) downwards along the distraction tube at a speed of 10 mm/minute until the fixed and moveable base plates contacted one another, representing the configuration of the distractor before activation. The springs were compressed by 9.1 mm or 60.67% of coil compression before being gradually released to return to their original length at a speed of 2 mm/minute (Figure 4B,C).

### 2.4. Statistical Analysis

The data were analyzed in the Statistical Package for Social Sciences software (SPSS; Version 18, IBM Corp., Chicago, IL, USA). Shapiro–Wilk’s tests showed a normal distribution of the data. Independent-samples *T*-tests were carried out to assess the differences between groups with a *p*-value < 0.05.

## 3. Results

### 3.1. Uniaxial Tensile Testing of Porcine Attached Gingiva

The average thickness of the buccal and lingual attached gingiva specimens was 1.87 ± 0.16 and 1.29 ± 0.12 mm, respectively. From the load–deflection curve of the uniaxial tensile tests, the mean failure load of BAG (17.84 ± 4.57 N) was significantly greater than LAG (10.44 ± 1.24 N) (*p* < 0.05) (Table 2).

The Young modulus of BAG (28.43 ± 6.51 MPa) was significantly higher than LAG (14.62 ± 1.88 MPa) (*p* < 0.05), but the tensile strength was not significantly different between groups (*p* > 0.05).

**Figure 5:**
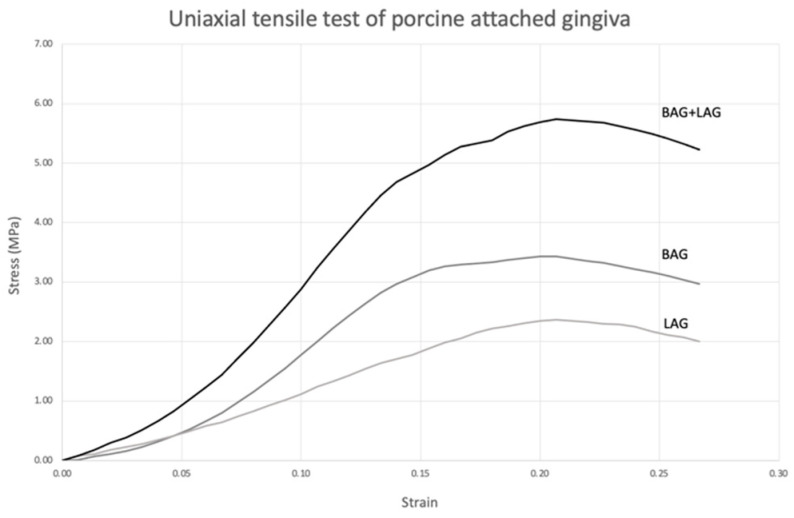
Representative stress–strain curves of porcine buccal and lingual attached gingiva from uniaxial tensile testing.

The stress–strain graph showed a non-linear J-shaped curve, where a gradual increase in tensile force until tissue failure was seen, demonstrating the characteristic mechanical behavior of connective tissue (Figure 5).

The upper curve in the stress–strain graph represents the combined response of BAG and LAG as clinically, both sides of attached gingiva would be stretched simultaneously during DO.

### 3.2. Compression and Extension Testing of NiTi Open-Coil Springs

**Figure 6:**
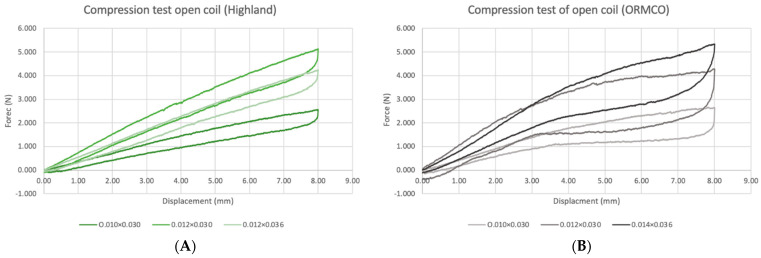
Load–displacement curves of NiTi open-coil springs of different manufacturers and dimensions. (**A**) The Highland group; (**B**) the ORMCO group.

The load–displacement curves for NiTi open-coil springs from two manufacturers with different dimensions are shown in Figure 6.

Springs were compressed to 55% of the initial length of 15 mm. At 8 mm of activation, the 0.010 × 0.030″, 0.012 × 0.030″, and 0.012 × 0.036″ Highland springs delivered 2.55 N5.12, and 4.24 N of force, respectively. The 0.010 × 0.030″, 0.012 × 0.030″, and 0.014 × 0.036″ ORMCO springs produced 2.63, 4.27 and 5.34 N of forces, respectively. For springs of the same dimensions, the magnitude of force generated by ORMCO springs tended to be greater than the Highland springs. Regardless of manufacturer, with the lumen diameter being constant, springs of greater wire diameter produced higher levels of constant force, as seen between the 0.010 × 0.030″ and 0.012 × 0.030″ groups. An inverse relationship was seen between lumen size and force levels instead, where the Highland 0.012 × 0.030″ springs had lower forces than the 0.012 × 0.036″ group with larger lumen diameter.

Upon deactivation, a rapid drop in force occurred between 8 and 7.7 mm. From 7.7 mm onwards, the Highland springs showed a more gradual reduction in the unloading force, similar to that of stainless-steel springs [14]. Conversely, the ORMCO spring displayed a constant force level until approximately 3 mm of displacement, after which another drop to the original levels occurred.

### 3.3. Compression and Extension Testing of NiTi Open-Coil Springs in the Novel Alveolar Distractor

**Figure 7:**
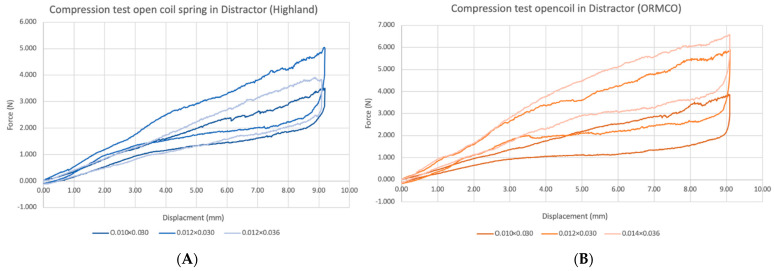
Load–displacement curves of NiTi open-coil springs of different manufacturers and dimensions tested in the novel alveolar distractor. (**A**) The Highland group; (**B**) the ORMCO group.

NiTi open-coil springs were inserted into the distraction tube and compressed by 9.1 mm from the original length of 15 mm, simulating the distractor configuration in the latency phase of DO before activation. The force–displacement curves of NiTi open-coil springs in the distractor are presented in Figure 7.

At 9.1 mm of activation, 0.010 × 0.030″, 0.012 × 0.030″, and 0.012 × 0.036″ Highland springs delivered 3.51, 5.01, and 3.80 N of force, respectively. The 0.010 × 0.030″, 0.012 × 0.030″, and 0.014 × 0.036″ ORMCO springs in the distractor produced 3.87, 5.88, and 6.58 N of force, respectively. The same trend of varying force levels from different wire and lumen diameters were seen. For springs from both manufacturers, upon unloading, the magnitude of force was found to rapidly decrease before producing a more constant force between 8.8 and 3 mm. From 3 mm onwards, the force levels gradually dropped again to zero or negative values.

A comparison of the unloading forces from the springs of the same manufacturer and dimensions tested with and without the distractor is shown in Table 3.

The 0.010 × 0.030″ springs from the Highland group produced significantly higher unloading forces at 0 and 4 mm of extension (*p* < 0.05) when tested in the distractor. On the contrary, the unloading forces from the remaining two spring dimensions for the Highland group were significantly lower at most displacements when placed in the distractor, except for 6 mm displacement of the 0.012 × 0.030″ group.

In the ORMCO group, the unloading forces that were significantly different were always higher during testing in the distractor irrespective of spring dimension and displacement distance. The unloading forces of 0.010 × 0.030″ and 0.014 × 0.036″ ORMCO springs in the distractor were only significantly higher than their counterparts at a distance of 0 mm, while the 0.012 × 0.030″ ORMCO group showed significant differences over more distances of 0, 2, and 4 mm

Comparing between the unloading forces from NiTi open-coil spring in the distractor and forces from tensile testing of porcine oral tissues presented in Table 4. All sized of springs with two manufacturer produced significant difference higher force at the beginning of distraction than forces from the stretch tissue of porcine attached gingiva. Additionally, then, forces from springs decreased significant different lower than forces delivered by tensile test of porcine attached gingiva, which increased in force along with the stretch of the tissues at 2, 4 and 6 mm.

**Figure 8:**
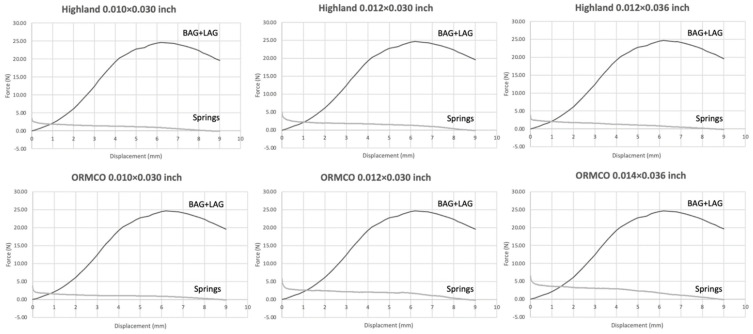
Combined load–displacement curves from the tensile tests of porcine attached gingiva with the unloading forces of the NiTi open–coil springs of different manufacturers and dimensions.

Load–displacement curves of the porcine attached gingiva were combined with those of the NiTi open-coil springs to estimate if the force levels from the springs could overcome the tensile forces of the soft tissues during alveolar distraction (Figure 8).

All three dimensions of the Highland springs produced unloading forces greater than the tensile forces of the attached gingiva over a minimum distance of 1 mm. The ORMCO springs tended to have the adequate force levels over slightly greater distances ranging from 1.0 to 1.5 mm

## 4. Discussion

The principle behind our novel alveolar distractor prototype using superelastic NiTi open-coil springs was to generate light and constant forces instead of heavy forces from a conventional distractor that relied on manual activation. Therefore, our experiment was designed to evaluate the ability of the NiTi springs in our prototype to generate forces that to overcome the mechanical strain of attached gingiva during the process of distraction.

Due to the morphological and biological resemblances to human oral soft tissues, porcine oral soft tissues were chosen to represent the biomechanical response to distraction forces [18]. A previous study used Thiel-embalmed porcine and human gingival tissues to investigate the tensile properties of the attached gingiva, buccal mucosa, and hard palatal mucosa [19,20]. They discovered that the ultimate tensile strength of human oral mucosa tissue that ranged from 1.00 to 3.80 MPa was comparable to that of porcine oral mucosa, which ranged from 1.06 to 3.94 MPa.

Gingival tissue comprises free gingiva, attached gingiva, and alveolar mucosa. Attached gingiva can withstand deformative forces from mastication because of its structure that consists of highly keratinized stratified squamous epithelium with more abundant collagenous fibers within the connective tissue layer. This results in higher stiffness and resistance to mechanical stress compared to alveolar and buccal mucosa, which have a larger proportion of non-keratinized epithelial tissue [21,22]. Goktas et al. [6] found that the biomechanical behavior of oral soft tissues is a function of their structure and location. They reported that the attached gingiva showed the highest failure load (19.74 ± 5.04 N), the ultimate tensile strength (3.94 ± 1.19 MPa) and the Young modulus (19.75 ± 6.20 MPa) compared to other types of porcine oral soft tissue. Our tests produced similar values, besides showing that the BAG of porcine oral tissues had a greater failure load (17.84 ± 4.57 N), the tensile strength (3.15 ± 0.64 MPa), and the Young modulus (28.43 ± 6.51 MPa) than the LAG.

The experimental use of superelastic NiTi springs in DO has been explored by several authors. In their rabbit model studies, Zhou et al. and Idelsohn et al. demonstrated the formation of new mineralized bone tissue across the distraction gap, confirmed by radiographs and histology [23,24]. However, their technique requires two surgical operations, one for the segmental mandibulectomy, and another for implanting the closed-coil springs after a latency period. Additionally, the action of the springs became impeded by the formation of granulation tissue around the springs, thus producing less force than expected.

Therefore, our novel alveolar distractor was designed to improve upon the concepts from previous studies. Our device is able to exert a relatively constant force over a range of 1 to 20 mm without the need of a second operation. We also added the distraction tube to protect the NiTi springs from tissue in-growth. In our study, we tested commercially available NiTi open-coil springs from two different manufacturers in three levels of force. From compression tests of the springs placed on a cylindrical rod as a central axle, all springs demonstrated a constant unloading force, which can be attributed to their superelastic properties. The ORMCO springs showed better efficiency in producing constant force levels than the Highland springs, where the springs with the largest dimensions of 0.014 × 0.036″ generated the highest magnitude of force in both the loading and unloading curves.

When the compression tests were carried out on the springs inserted in the distraction device without a central axle, the loading and unloading forces of the spring were indirectly measured by those produced by the head of the movable plate. Our results showed that a majority of the forces measured in this experimental setup were significantly lower. In this system, friction could arise when the head of the plate slides along the distraction tube, as some parts of the compressed springs would bow and contact the inner wall of the distraction tube. Hence, springs with diameters more identical to the inner diameter of the distraction tube would be more favorable as there would be less tolerance for the springs to bend out of shape and contact the tube wall. This would reduce the risk of friction that results in diminished unloading forces from the elongating springs [25]. Although increasing diameter would decrease force levels, the range of superelastic activity would be extended. Besides lumen diameter, a balance of other factors such as wire diameter and pitch should be taken into consideration when selecting the appropriate springs for any device. These can affect the force levels and superelastic range of the springs [26].

The regenerative capacity of bone is influenced by the rate and frequency of distraction. In an animal study by Kessler et al., continuous distraction with low forces was more rapid and effective compared to intermittent activations with heavy forces. The former protocol produced mature bone by intramembranous ossification while the latter resulted more in chondroid ossification or cartilage formation [27]. Nevertheless, the magnitude of force should be high enough to exceed the opposing restraint from gingival tissue for the bone segments to be separated over the desired distance. Thus, the distinctive point of our prototype of the distractor over the conventional type are less patient compliance, less pain and a faster regenerative process due to providing light and continuous force, moving bone segments in a desired vector, without distortion on activation and increasing the rate of success of treatment [10,23].

The forces from the NiTi springs in our study appear to be able to overcome the tensile forces of the porcine tissues over a distance of 0.75 to 1.50 mm. This value should be interpreted with caution, as our study was not performed in living tissue, so the actual physiological response to the distraction forces could differ. As tissue remodeling takes place from the moment distraction forces are applied, this would gradually release the initial tissue tension that occurs. The rate of distraction for 1 to 2 mm per day was suitable without inadequate bone formation and non-union in faster rate and bone union in slower rate [2]. Moreover, the primary mechanical resistance from oral soft tissues has been found to come from the mandibular periosteum. Debelmas et al., who conducted tensile tests for periosteal tissue harvested from human cadavers, reported a Young modulus of 10.75 MPa [28]. This value was 3-fold lower than that of porcine mandibular periosteum reported in another study [29]. Hence, our study, which used the stiffer porcine oral soft tissues, could have underestimated the distance over which the NiTi open-coil springs could overcome the soft tissue forces.

From our results, the 0.014 × 0.036″ ORMCO springs would be the preferred option for our novel distractor design as they could produce the highest unloading force over the longest distance. Future in vivo studies would certainly be valuable to explore the living tissue response to our novel distraction device.

## 5. Conclusions

This preliminary in vitro study demonstrated the design of a novel alveolar distractor that uses NiTi open-coil springs to produce light, constant, and continuous forces, which are beneficial for DO to eliminate the reliance on patient cooperation and could theoretically lead to bone regeneration without damaging the gingival soft tissue. Proper selection of NiTi spring dimensions is necessary to produce the optimum levels of unloading force for effective distraction and bone regeneration.

## Figures and Tables

**Table 1:** Dimensions of commercial NiTi open-coil springs obtained for mechanical testing.

Company	Force Level	Wire Diameter (Inch)	Lumen Size (Inch)	Ref No.	Lot No.	Initial Length (mm)	No. of Specimens
Highland	Light	0.010	0.030	11100105414	676491	15	10
	Medium	0.012	0.036	11100125424	630782	15	10
	Heavy	0.012	0.030	11100125414	528294	15	10
ORMCO	Light	0.010	0.030	221-5510	8H89	15	10
	Medium	0.012	0.030	221-5512	18B70	15	10
	Heavy	0.014	0.036	221-5514	15E118	15	10

**Table 2:** Tensile properties of porcine buccal and lingual attached gingiva.

Properties	Buccal Attached Gingiva		Lingual Attached Gingiva		*p*-Value
	Mean	S.D.	Mean	S.D.	
Failure Load (N)	17.84	4.57	10.44	1.24	0.02 *
Tensile Strength (MPa)	3.15	0.64	2.60	0.31	0.14
Young Modulus (MPa)	28.43	6.51	14.62	1.88	0.01 *

* Statistically significant difference at *p* < 0.05.

**Table 3:** Comparison of the mean load between NiTi springs tested with and without the novel alveolar distractor.

Company and Spring Dimensions	Distance (mm.)	NiTi Spring Only		NiTi Spring in Distractor		*p*-Value
		Mean	S.D.	Mean	S.D.	
Highland 0.010 × 0.030″	0	2.55	0.06	3.40	0.26	<0.001 *
	2	1.45	0.15	1.46	0.07	0.921
	4	0.95	0.85	1.16	0.04	0.002 *
	6	0.42	0.16	0.53	0.07	0.180
Highland 0.012 × 0.030″	0	5.12	0.12	4.22	0.21	<0.001 *
	2	3.28	0.09	1.88	0.14	<0.001 *
	4	2.2	0.1	1.54	0.16	<0.001 *
	6	1.06	0.05	0.97	0.15	0.232
Highland 0.012 × 0.036″	0	4.24	0.18	3.56	0.36	0.005 *
	2	2.69	0.14	1.58	0.09	<0.001 *
	4	1.80	0.14	1.07	0.06	<0.001 *
	6	0.79	0.1	0.50	0.05	0.001 *
ORMCO 0.010 × 0.030″	0	2.63	0.15	3.38	0.15	<0.001 *
	2	1.25	0.15	1.16	0.03	0.237
	4	1.11	0.12	1.06	0.04	0.419
	6	0.57	0.12	0.65	0.03	0.203
ORMCO 0.012 × 0.030″	0	4.27	0.21	5.64	0.24	<0.001 *
	2	1.79	0.16	2.21	0.09	0.001 *
	4	1.55	0.23	1.94	0.03	0.019 *
	6	0.81	0.4	1.11	0.04	0.156
ORMCO 0.014 × 0.036″	0	5.34	0.19	6.06	0.37	0.005 *
	2	2.80	0.21	3.07	0.46	0.264
	4	2.28	0.07	2.29	0.25	0.888
	6	1.15	0.1	1.13	0.12	0.800

* Statistically significant difference at *p* < 0.05.

**Table 4:** Comparison of the mean load of tensile test between forces from NiTi springs tested in the distractor and forces from porcine attached gingiva.

Company and Spring Dimensions	Distance (mm.)	Springs in Distractor		Porcine Attached Gingiva		*p*-Value
		Mean	S.D.	Mean	S.D.	
Highland 0.010 × 0.030″	0	3.51	0.22	2.14	0.84	0.007 *
	2	1.74	0.12	6.14	2.77	0.002 *
	4	1.62	0.12	19.25	6.73	0.004 *
	6	1.25	0.59	24.48	5.48	0.001 *
Highland 0.012 × 0.030″	0	5.01	0.15	2.14	0.84	<0.001 *
	2	2.23	0.08	6.14	2.77	0.034 *
	4	2.01	0.07	19.25	6.73	0.005 *
	6	1.64	0.15	24.48	5.48	0.001 *
Highland 0.012 × 0.036″	0	3.81	0.19	2.14	0.84	0.002 *
	2	2.04	0.59	6.14	2.77	0.03 *
	4	1.76	0.09	19.25	6.73	0.004 *
	6	1.19	0.07	24.48	5.48	0.001 *
ORMCO 0.010 × 0.030″	0	3.86	0.17	2.14	0.84	0.002 *
	2	1.55	0.06	6.14	2.77	0.021 *
	4	1.34	0.07	19.25	6.73	0.004 *
	6	1.1	0.04	24.48	5.48	0.001 *
ORMCO 0.012 × 0.030″	0	5.88	0.23	2.14	0.84	<0.001 *
	2	2.63	0.06	6.14	2.77	0.047 *
	4	2.45	0.05	19.25	6.73	0.005 *
	6	2.01	0.04	24.48	5.48	0.001 *
ORMCO 0.014 × 0.036″	0	6.58	0.41	2.14	0.84	<0.001 *
	2	3.61	0.56	6.14	2.77	0.011 *
	4	3.24	0.46	19.25	6.73	0.006 *
	6	2.63	0.34	24.48	5.48	0.001 *

* Statistically significant difference at *p* < 0.05.

## Data Availability

The data are available upon request from the corresponding author.

## References

[1] McCarthy J.G., Stelnicki E.J., Mehrara B.J., Longaker M.T. (2001). Distraction Osteogenesis of the Craniofacial Skeleton. Plast. Reconstr. Surg..

[2] Natu S.S., Ali I., Alam S., Giri K.Y., Agarwal A., Kulkarni V.A. (2014). The Biology of Distraction Osteogenesis for Correction of Mandibular and Craniomaxillofacial Defects: A Review. Dent. Res. J..

[3] Cope J.B., Samchukov M.L., Cherkashin A.M. (1999). Mandibular Distraction Osteogenesis: A Historic Perspective and Future Directions. Am. J. Orthod. Dentofac. Orthop..

[4] Chin M., Toth B.A. (1996). Distraction Osteogenesis in Maxillofacial Surgery Using Internal Devices: Review of Five Cases. J. Oral. Maxillofac. Surg..

[5] Peacock Z.S., Tricomi B.J., Lawler M.E., Faquin W.C., Magill J.C., Murphy B.A., Kaban L.B., Troulis M.J. (2014). Skeletal and Soft Tissue Response to Automated, Continuous, Curvilinear Distraction Osteogenesis. J. Oral Maxillofac. Surg..

[6] Goktas S., Dmytryk J.J., McFetridge P.S. (2011). Biomechanical Behavior of Oral Soft Tissues. J. Periodontol..

[7] Andrade N., Gandhewar T., Kalra R. (2011). Development and Evolution of Distraction Devices: Use of Indigenous Appliances for Distraction Osteogenesis-An overview. Ann. Maxillofac. Surg..

[8] Agarwal R. (2013). Unfavourable Results with Distraction in Craniofacial Skeleton. Indian. J. Plast. Surg..

[9] Ettl T., Gerlach T., Schüsselbauer T., Gosau M., Reichert T.E., Driemel O. (2010). Bone resorption and complications in alveolar distraction osteogenesis. Clin. Oral Investig..

[10] Saulacić N., Martín M.S., Camacho M.D.L.A.L., García A.G. (2007). Complications in Alveolar Distraction Osteogenesis: A Clinical Investigation. J. Oral Maxillofac. Surg..

[11] Mofid M.M., Manson P.N., Robertson B.C., Tufaro A.P., Elias J.J., Vander Kolk C.A. (2001). Craniofacial Distraction Osteogenesis: A Review of 3278 Cases. Plast. Reconstr. Surg..

[12] Es-Souni M., Es-Souni M., Fischer-Brandies H. (2005). Assessing the Biocompatibility of NiTi Shape Memory Alloys used for Medical Applications. Anal. Bioanal. Chem..

[13] Thompson S.A. (2000). An Overview of Nickel-Titanium Alloys Used in Dentistry. Int. Endod. J..

[14] Shimoga G., Kim T.-H., Kim S.-Y. (2021). An Intermetallic NiTi-Based Shape Memory Coil Spring for Actuator Technologies. Metals.

[15] Bourke A., Daskalogiannakis J., Tompson B., Watson P. (2010). Force Characteristics of Nickel-Titanium Open-Coil Springs. Am. J. Orthod. Dentofacial Orthop..

[16] Celesti C., Gervasi T., Cicero N., Giofrè S.V., Espro C., Piperopoulos E., Gabriele B., Mancuso R., Lo Vecchio G., Iannazzo D. (2022). Titanium Surface Modification for Implantable Medical Devices with Anti-Bacterial Adhesion Properties. Materials.

[17] Sidambe A.T. (2014). Biocompatibility of Advanced Manufactured Titanium Implants—A Review. Materials.

[18] Heaney T.G. (1977). A Histological Investigation of the Influence of Adult Porcine Gingival Connective Tissues in Determining Epithelial Specificity. Arch. Oral Biol..

[19] Okada R., Tsunoda A.T.S.U.N.O.B.U., Momiyama N.A.O.K.O., Kishine N., Kitamura K., Kishimoto S.E.I.J.I., Akita K. (2012). Thiel’s Method of Embalming and Its Usefulness in Surgical Assessments. Nihon Jibiinkoka Gakkai Kaiho.

[20] Choi J.J.E., Zwirner J., Ramani R.S., Ma S., Hussaini H.M., Waddell J.N., Hammer N. (2020). Mechanical Properties of Human Oral Mucosa Tissues Are Site Dependent: A Combined Biomechanical, Histological and Ultrastructural Approach. Clin. Exp. Dent. Res..

[21] Nakamura M. (2018). Histological and immunological characteristics of the junctional epithelium. Jpn. Dent. Sci. Rev..

[22] Bassols A., Costa C., Eckersall P.D., Osada J., Sabria J., Tibau J. (2014). The Pig as an Animal Model for Human Pathologies: A Proteomics Perspective. PROTEOMICS–Clin. Appl..

[23] Zhou H.Z., Hu M., Yao J., Ma L. (2004). Rapid Lengthening of Rabbit Mandibular Ramus by Using Nitinol Spring: A Preliminary Study. J. Craniofacial Surg..

[24] Idelsohn S., Pena J., Lacroix D., Planell J.A., Gil F.J., Arcas A. (2004). Continuous Mandibular Distraction Osteogenesis Using Superelastic Shape Memory Alloy (SMA). J. Mater. Sci. Mater. Med..

[25] Stiernstedt J., Rutland M., Attard P. (2005). A Novel Technique for the In Situ Calibration and Measurement of Friction with the Atomic Force Microscope. Rev. Sci. Instrum..

[26] Miura F., Mogi M., Ohura Y., Karibe M. (1988). The Super-Elastic Japanese Niti Alloy Wire for Use in Orthodontics Part III. Studies On the Japanese Niti Alloy Coil Springs. Am. J. Orthod. Dentofac. Orthop..

[27] Kessler P., Neukam F.W., Wiltfang J. (2005). Effects of Distraction Forces and Frequency of Distraction on Bony Regeneration. Br. J. Oral Maxillofac. Surg..

[28] Debelmas A., Picard A., Kadlub N., Boisson J. (2018). Contribution of the Periosteum to Mandibular Distraction. PLoS ONE.

[29] Popowics T., Zhu Z., Herring S. (2002). Mechanical Properties of the Periosteum in the Pig, Sus Scrofa. Arch. Oral Biol..

